# Contribution of health information system to child immunization services in Ethiopia: baseline study of 33 woredas

**DOI:** 10.1186/s12911-022-01796-8

**Published:** 2022-03-11

**Authors:** Abebaw Worku, Hibret Alemu, Hiwot Belay, Afrah Mohammedsanni, Wubshet Denboba, Frehiwot Mulugeta, Shemsedin Omer, Biruk Abate, Mesoud Mohammed, Mohammed Ahmed, Yakob Wondarad, Meskerem Abebaw

**Affiliations:** 1 Ethiopia Data Use Partnership, Addis Ababa, Ethiopia; 2 Ethiopia Ministry of Health, Addis Ababa, Ethiopia

**Keywords:** Ethiopia, Immunization coverages, Health information system

## Abstract

**Background:**

Monitoring progress using appropriate data, with a functional health information system (HIS), believed to be very crucial for success of immunization program. Baseline study was conducted to assess, immunization service coverage, HIS performance status, and their relationships.

**Methods:**

A linked facility and population-based survey was conducted concurrently from September 21 to October 15, 2020. A total of 3016 households were reached to interview mothers having children aged 12–23 months in the 33 woredas. Overall 81 health posts, 71 health centers, and 15 hospitals were selected for the facility survey. The study used modified Performance of Routine Information System Management (PRISM) tools for the facility survey and a structured questionnaire for the household survey. Using STATA 14.0 software, mixed effect modeling was employed to control the effect of clustering and potential confounders.

**Results:**

The proportion of fully immunized children was 58%. Coverages of measles (at least one dose) and penta3 immunization (received all 3 doses of DPT-HepB-Hib vaccine) were 86%, and 85% respectively. About 27% of mothers had missed their child immunization card mainly due to misplacing or lost. Except ‘source document completeness’ (85%) and ‘use of data for planning and target setting’ (84%), other data quality and use indicators like ‘data accuracy’ (63%), ‘data use for performance review and evidence-based decision making’ (50%), and ‘data use to produce analytical reports’ (31%) show low performance. The odds of fully immunized children is 37% lower in Muslims compared with Orthodox Christians (AOR, 0.63; 95%CI: 0.46, 0.88), higher by 42% with father’s secondary education compared with no education (AOR, 1.42; 95%CI: 1.05, 1.92), and highest wealth quintile compared with lowest quintile (AOR, 2.49; 95%CI: 1.54, 4.03). For each additional score of HIS infrastructure availability, the odds of fully immunized children increased by 22% (AOR:1.22; 95% CI: 1.03, 1.44).

**Conclusions:**

Child immunization coverages are promising However, the current HIS performance is suboptimal. Both service user and HIS related factors are important for immunization service uptake. Documenting required information and advising mothers to keep immunization cards by health workers, and working to have functional HIS are recommended.

**Supplementary Information:**

The online version contains supplementary material available at 10.1186/s12911-022-01796-8.

## Background

In many countries, vaccine-preventable diseases remain major causes of child mortality. Over 10% of children under one year old in developing countries were not receiving even one dose of DPT vaccine, compared with 2% in industrialized countries [1]. It has been estimated that almost 18 million infants have not received the first dose of DPT, one third of them in Africa [2]. It has been also recognized that vaccine preventable diseases are responsible for 16% of under-five mortality in Ethiopia [3].

The Ethiopian Demographic Health Survey (EDHS) 2016 report revealed that 38.5% children age 12–23 months have received all eight basic vaccinations—one dose each of BCG and measles and three doses each of DPT-HepB-Hib and polio vaccine [4]. The coverage was 24.3% in 2011 EDHS [5]. Based on the recent Mini-EDHS, fully vaccinated children reached to 44.1% [6] which was only 14.3% in 2000 [7].

Variety of factors affect utilization of immunization services. Further analysis of the 2019 EDHS data indicated that urban children are more likely to be fully immunized than rural children (62% vs. 36%). Variations are also seen by educational status (41% in no education vs. 61% in secondary or higher), wealth quantile (26% in the lowest vs. 67% in the highest), and regions (lowest in Somali (19%) and highest in Addis Ababa (83%) [6]. The presence of significant association with urban residence, higher educational status, richer wealth quintile, employment and receiving ANC and delivery service were also observed in further analysis of 2016 EDHS [8].

Supply side factors including availability of equipment, supplies, medicine, health workers training, and availability of guidelines are the major gaps observed in health facilities of Ethiopia [9] which directly or indirectly affects service uptake and quality of immunization service.

Routine data quality, management and use are the key factors for monitoring service uptake and quality through regular review of routine health data and analysis of root causes. A systematic review of 12 studies identified three categories of factors including governance (leadership, participatory monitoring, regular review of data); production of information (presentation of findings, data quality, qualitative data); and health information system resources (electronic health management information systems, organizational structure, training) that influence the use of health service delivery indicators to improve delivery of primary health care services in low- and middle-income countries [10].

HIS interventions helped for improving data quality and information use for key public health programs like immunization [11]. Nonetheless, studies from Ethiopia and elsewhere showed that the quality of routine data gathered in the health system is poor [12, 13]. Quality of immunization data in other countries like Ghana is characterized by underreporting and over reporting (underreporting for BCG and over reporting for penta and measles vaccinations) [14]. The proportion of routine health information utilization for decision making in Ethiopia is low [15]. Most of the primary health facilities are not using their data for monitoring their activities routinely [16].

Ensuring access, and maintaining quality of service requires day-to-day follow up and timely informed decisions [17] which is not possible without functional Health Information System (HIS). Ethiopia is introducing new initiative (HIS transformation plan) to transform routine health information and improve the quality and use of data to address the essential health needs of the population. As part of the initiative, partners, universities and the Ministry of Health are working together. Improving health care services through enhanced health information system using human capacity development model is one of the ongoing program in Ethiopia [18]. Demonstration/learning woredas, with their health facilities, are also selected for intense support and HIS intervention in order to scale up best practices. Interventions include; need based trainings, supportive supervision, regular mentorship and material support. Therefore, this baseline survey, was planned to assess current immunization service coverage and measure its relationship with HIS performance of nearby health facilities. Later on, when the end line survey conducted, the effect of interventions will be evaluated.

## Methods

### Study design

A linked facility and population-based survey was conducted to assess the stated objectives. Similar survey will be conducted at the end line. One of the major focus of household level survey was to assess immunization coverage. A simultaneous facility level survey was conducted, focusing on HIS infrastructure and HIS performance (data quality, management and use), in facilities that serve the selected kebele populations.

### Study area and period

This study covers 33 woredas from all regions of Ethiopia. At least one woreda (district) per region was included. The selection of the districts and facilities for monitoring outcomes based on the planned HIS interventions. The data collection period was from September 21 to October 15, 2020.

### Target population and sampling

The target population for this study was women with children 12–23 months of age. A two-stage sampling technique was applied to select the sample population. At first stage, 102 clusters (kebeles) were selected from 33 woredas. At second stage, the sampling frame of the target population was prepared after census in each kebele. Then, 29 targeted households were selected from each kebele using systematic random sampling technique (every other targeted households). A total of 3,016 households with targeted mothers were included.

For the facility survey, health posts and health centers providing services for the catchment population in selected kebeles were included in the sample along with all hospitals found in the 33 woredas. Overall, 81 health posts, 71 health centers, and 15 hospitals were selected for the facility survey. However, 74 health facilities (70 health centers and four hospitals) data were linked with 3,016 households for the regression analysis.

### Data collection and procedures

A structured data collection tool was adapted from the demographic and health survey questionnaire [19] and designed on SurveyCTO (software tool for data collection) to collect the population-based data. Similarly, modified Routine Information System Management (PRISM) tools, designed on SurveyCTO, were used for the facility survey. In order to assure the quality of the data; the survey team received an intensive 5-day training using a structured training manual, the questionnaires and the SurveyCTO program pretested before the start of data collection (all the data collectors and supervisors were part of the pretesting), daily data quality checks carried out as data uploaded to the server, and all indicators calculated according to the tabulation plan set out during the planning phase, including correct treatment of ‘don’t know’ responses. Survey team leaders and regional coordinators supervised data collection.

Data collectors and supervisors (minimum of degree holders) who had experience collecting survey data were deployed and used tablets to collect data.

### Data analysis

Data use and data quality scores were generated based on the PRISM tool. Mixed effect modeling (multi-level logistic regression) was employed to account for clustering at the health facility level as well as controlling all potential confounders at different levels (both at health facility and individual service user levels). Different data sets produced from the household and health facility survey were carefully cleaned and linked to perform regression analysis. We used Stata version 14.0 for the statistical analysis. The outcome variable (full immunization) has binary categories with “No” and “Yes”. “Complete or full immunization” coverage is defined as a child that has received one dose of BCG, three doses of pentavalent, pneumococcal conjugate (PCV), oral polio vaccines (OPV); two doses of Rota virus and one dose of measles vaccine. After running the null model, intraclass correlation coefficient (ICC) was estimated to know the percentage of total variation explained by variation between health facilities. Finally, individual and group level variables were checked and entered to produce the last model. Potential explanatory variables at the individual level includes residence, religion, marital status of mother, educational status of mother & partner/husband, main occupation of mother/partner, wealth index (computed from 10 asset variables), and household distance from health facilities (in kilo meters). Similarly, four categories of facility level explanatory variables including HIS infrastructure, data quality, data use and data management were considered for the analysis. As mentioned in the operational definitions, most of the health facility level variables were composite indices with averaged scores (see Additional file 1).

## Results

### Background characteristics of study participants

More than half (53%) of the participants were 20 to 30 years old. One third (33%) didn’t attend formal education. Few (5%) mothers were employed in government or private institutions whereas better percentage (16%) of their partners were employed (Table 1).

**Table 1:** Frequency and percentage of respondents/mothers by their background characteristics: Ethiopia immunization study, 2020

Maternal characteristics	Frequency (n)	Percent
Residence (region cluster)		
Emerging regions (pastoral and agro-pastoral)	580	19.20
Agrarian regions	2030	67.30
Urban	406	13.50
Total	3016	100
Age of mother (years)		
15–19	168	5.62
20–24	677	22.66
25–29	913	30.56
30–34	614	20.55
35–39	504	16.87
40–44	96	3.21
45–54	16	0.54
Total	2988	100
Age of her child (months)		
12–17	1622	53.78
18–23	1394	46.22
Total	3016	100
Currently married or partnered		
Yes	2832	93.90
Total	3016	100
Religion		
Orthodox	1288	42.71
Muslim	1261	41.81
Protestant	424	14.06
Others (Catholic, traditional)	43	1.43
Total	3016	100
Mothers educational status		
No education	991	32.86
Elementary (1–8)	1388	46.02
Secondary	469	15.55
Above secondary	168	5.57
Total	3016	100
Partner educational status		
No education	712	25.14
Elementary (1–8)	1208	42.66
Secondary	599	21.15
Above secondary	313	11.05
Total	2832	100
Mothers occupational status		
Employed	154	5.11
Farmer	864	28.65
Merchant	185	6.13
Unemployed	1577	52.29
*Others	236	7.82
Total	3016	100
Partner occupational status		
Employed	497	17.55
Farmer	1480	52.26
Merchant	153	5.40
Unemployed	54	1.91
*Others	648	22.88
Total	2832	100

^*^Students, daily laborer, retired, too young to work, attends to home chore

### Child immunization service

#### Availability of immunization card

Child immunization cards were available in 61.3% of the eligible households. It is also reported but missed immunization cards in 27.3% of eligible households. The main reason for missing immunization card is misplacing or lost (Table 2).

**Table 2:** Availability of immunization card and listed reasons for missing cards, Ethiopia, 2020

Variables	Frequency	Percent
No card/notebook	344	11.41
Yes, card/notebook available	1849	61.31
Yes, but card missed	823	27.29
Total	3016	100
Reasons of missing immunization cards (multiple response)		
Misplaced/lost card	423	36.25
Destroyed card	160	13.71
Never received card	223	19.11
Card in home, but inaccessible (i.e. lo	157	13.45
Card not available or outside of home	106	9.08
Card kept at health facility	78	6.68
*Other	20	1.71
Total	1167	100

*At mother’s family home, come from other kebele, card lost at health facility, card not given

### Child immunization coverage based on specific vaccine types

The immunization coverage indicated that 96% of children have history of BCG immunization. The coverage is also high (91%) based on valid evidences using cards and BCG scar. Similarly, based on valid evidence from immunization card, proportion of children who received all required (the three) doses of vaccinations is 90% for OPV, 85% for pentavalent (DPT-HepB-Hib), and 85.6% for PCV. Proportion of children who received two or more dose of rota virus vaccination is 86.8%. Immunization coverage of children with at least one dose measles vaccination is 86.4% and coverage of vitamin A supplement is 80.44%. Proportion of targeted children (12–23 months age) who received all required doses of routine immunization (BCG, opv3, pcv3, penta3, rota2, and measles) based on valid evidence (card or scar) is 57.6% (Table 3).

**Table 3:** Immunization coverage of children (12–23 months age) based on specific type of vaccine, Ethiopia, 2020

Vaccine types	Frequency	%	95% CI
Crude BCG vaccine coverage (n = 2933)	2816	96.01	95.24–96.67
Valid BCG vaccine coverage (n = 2736)	2489	90.97	89.84–91.99
OPV0 (n = 2471)	1548	62.65	62.65–64.53
OPV1 (n = 2057)	1901	92.42	91.12–93.48
OPV2 (n = 2260)	2059	91.11	89.86–92.21
OPV3 (n = 2629)	2370	90.15	88.95–91.23
DPT-HepB-Hib1 (n = 2159)	1925	89.16	87.78–90.41
DPT-HepB-Hib2 (n = 2387)	2109	88.35	87.0–89.58
DPT-HepB-Hib3 (n = 2586)	2203	85.19	83.77–86.51
PCV1 (n = 2134)	1919	89.93	88.57–91.13
PCV2 (n = 2379)	2112	88.78	87.44–89.98
PCV3 (n = 2561)	2192	85.59	84.18–86.9
Rota1 (n = 2274)	1948	85.66	84.16–87.05
Rota 2 + (n = 2878)	2497	86.76	85.47–87.95
At least one dose of measles (MCV 1 +) (n = 3016)	2606	86.41	85.13–87.58
Vitamin A supplement (n = 3016)	2426	80.44	78.98–81.82
Fully immunized (Received all the required doses, BCG, opv3, pcv3, penta3, rota2, measles) (n = 3016)	1736	57.56	55.79–59.31

### HIS resources availability and HIS performance

The average score of availability of key HIS infrastructure at facility level was about 82% and the average score of availability of functional eHIS tools including EMR, DHIS2, eCHIS, and HRIS was 39%. Functional DHIS2 was available in most of the health facilities. An average of 8 staffs per health facility received HIS related trainings including basic HMIS, data quality, data use, data analysis, eHIS tools and other related trainings, and 72% of health facilities have at least one trained staff in any of HIS topics (Table 4).

**Table 4:** HIS resource and performance of health facilities in the 33 woredas based on selected HIS indicators, Ethiopia 2020

Selected HIS indicators	Percentage (score)	95% CI
HIS resource		
HIS infrastructure at facility level (average score)	82	81–83
eHIS tools availability score	39	38–40
Number of staff received training on HIS topics at facility level one-year prior the survey (average score)	7.8	7.4–8.1
Percentage of facilities which have at least one trained staff in any of HIS topics	72	70–74
Data management		
Data quality control practices (average score)	72	71–74
Level of data analysis practice (average score)	68	67–69
Data visualization practice	86	84–87
Presence of feedback mechanism	92	90–93
Data quality		
Source document completeness: all three months complete	85	83–86
Data accuracy: acceptable range (90%–110%)	63	61–65
Data accuracy: over reporting (< 90%)	16	15–18
Data accuracy: under reporting (> 110%)	30	28–31
Data use		
Use of routine data for RHIS quality improvement (average score)	45	43–46
Use of routine data for performance review and evidence-based decision making (average score)	50	48–51
Use of data for annual plan and target setting	84	83–85
Use of data to produce narrative analytical reports	31	29–32

*Source document completeness and data accuracy calculation is based on pentavalent vaccination using three months report (April, May & June, 2020)

The average score of health facilities data quality control practice was 72%, and the average score of health facilities data analysis practice was 68%. About 86% of facilities produced data visuals (graphs, tables, maps, etc.) and showing achievements toward targets using raw HMIS data. About 92% of facilities received feedback on the reported HMIS data from the woreda or higher level. Pentavalent vaccine source document completeness of health facilities, verification of the quality in completing primary source documents (e.g. registers, patient records, etc.), was analyzed for each of the three months (April, May June, 2020). Health facilities with complete source document for all the three months was 85%. Staffing issues (shortage, absence), poor understanding of the data element by health workers and negligence were top three reasons for incomplete source document. Report accuracy was considered acceptable within a 10% tolerance range, meaning, if the data in the health facility monthly report is matching those of the source document between 90%-110% for each assessed indicator. Based on pentavalent vaccine three months (April, May June, 2020) report, majority (63%) of the health facilities met the acceptable report accuracy. However, significant number of facilities had under reporting (30%) and over reporting (16%). The main reasons cited for the observed discrepancies between the recorded data and report include: arithmetic error, data entry error, information from all source documents not compiled correctly, and lack of emphasis for data accuracy. The average score of health facilities using routine data to improve HMIS data quality was 45%, and the average score of health facilities using data for performance review and evidence-based decision making was 50%. About 84% of health facilities use routine data for annual plan and target setting, and 31% of health facilities had evidence of analytical report production using HMIS data (Table 4).

### Factors associated with immunization coverage

About 35% of the variation of children’s full immunization is explained by difference among health facilities, (ICC: 0.35; 95% CI: 0.27, 0.44). The odds of fully immunized children is 37% lower in Muslims compared with Orthodox Christians (AOR, 0.63; 95%CI: 0.46, 0.88) and higher by 42% with father’s secondary education compared with no education (AOR, 1.42; 95%CI: 1.05, 1.92). Significant association for full immunization was also observed in highest wealth quintile compared with lowest quintile (AOR, 2.49; 95%CI: 1.54, 4.03). For each additional score of HIS infrastructure availability, the odds of fully immunized children increased by 22% (AOR:1.22; 95% CI: 1.03, 1.44) (Table 5).

**Table 5:** Factors associated with full immunization of children, Ethiopia, 2020

Explanatory variables	Immunization coverage	AOR	P > z	[95% Conf. Interval]
Region cluster (residence)				
Emerging regions	81.7	1		1
Agrarian	87.6	1.49	0.29	0.71–3.16
Urban	97.9	1.88	0.24	0.66–5.37
Religion				
Orthodox Christian	91.3	1		1
Muslim	87.3	0.63	0.01	0.46–0.88*
Protestant	80.0	0.91	0.70	0.57–1.45
Others	94.4	0.47	0.10	0.19–1.17
Education of father				
No education	83.3	1		1
Elementary (1–8)	85.7	1.06	0.61	0.84–1.36
Secondary	94.1	1.42	0.02	1.05–1.92*
Above secondary	95.6	1.03	0.88	0.71–1.49
Wealth Index				
Lowest	78.5	1		1
Low	84.9	1.12	0.46	0.83–1.50
Middle	87.8	1.21	0.23	0.89–1.65
High	90.7	1.38	0.10	0.94–2.01
Highest	97.8	2.49	0.00	1.54–4.03*
Distance from HH to HF (mean 3.98 km)		0.98	0.19	0.94–1.01
HIS infrastructure availability score (82%)		1.22	0.02	1.03–1.44*
eHIS tools availability score (39%)		1.31	0.053	0.99–1.73
Use of routine data for performance review (50%)		0.66	0.18	0.36–1.21

*Significantly associated

## Discussion

The study outlines the immunization coverage status of Ethiopia among children 12–23 months old, HIS performance of health facilities, and their relationships. The study provided up to date information for the ongoing efforts by the government to achieve the set targets. The study revealed that most of immunization coverages by individual vaccines (BCG, OPV3, PCV3, Penta3, Rota2, Measles) is above 85% but the percentage of children who fully immunized (receiving all the required doses) is 57.6%. The immunization coverage in this study is higher than the 2019 Mini EDHS coverage report (44.1%) of Ethiopia [6], and pooled estimates (47%) of different studies [20]. Similarly, it is higher than DHS coverage reports of sub Saharan countries such as Liberia (50.8%), Mali (44.6%), Nigeria (31.3%) and Uganda (55.2%) [19]. However, it is lower than recent DHS reports from Ghana (77.3%), Senegal, Kenya (71.1%), Mozambique (65.8%), Malawi (75.8%), Rwanda (92.6%), Tanzania (75.3%), and Zambia (75%) [19]. The finding indicates significant change in the trends of vaccine coverage in Ethiopia but it is far from the target (95%) set by Health Sector Transformation Plan (HSTP) of the country [21]. The presence of documentation problems on the observed immunization cards and missing of many immunization cards (27.3%) can be among the major reasons for lower estimation of valid coverages observed in this study.

The study showed that the HIS resource availability scores of health facilities like HIS infrastructure (82%) is moderate. However, HIS training (only 8 staff per health facility) and eHIS tools availability score (39%) are very low. Evidence from systematic reviews indicated that moving from paper-based information systems to electronic and digital systems probably allows staff at healthcare facilities to collect some types of routine health information faster and with fewer mistakes [22]. Studies from Nigeria, South Africa and Ethiopia revealed that availability of functional eHIS tools and providing HIS training to use the tools, like DHIS2, are very crucial to improve data quality like document completeness and accuracy [23–27]. HIS training is also significantly associated with utilization of health information by health care workers [28]. The finding implies that interventions are needed for observed gaps in both availability of eHIS tools and training of staff on the tools.

Compared with other indicators of data management, level of ‘data analysis practice score’ is low (72%) which can be the reason for low ‘use of data to produce narrative analytical reports’ (31%), found in this study. Previous studies indicated that health data management practices of health professionals were found to be inadequate [29]. The finding indicates the need of improving data analysis skills of health care workers in the health facilities.

The study highlights that both data quality and data use indicators showed suboptimal performance compared with the national target of 90%. Indicators like ‘source document completeness’ (85%) and ‘use of data for annual plan and target setting’ (84%) are promising. However, other data quality and use indicators showed very low performance. Data accuracy (acceptable range) of health facilities is low (63%) but it is better than previous study from Ethiopia (48%) [30]. The current study showed that use of routine data for RHIS quality improvement score is also low (45%). This implies that due attention should be provided to improve data quality. The ‘use of data for performance review and decision making’ score is 50%. The finding is consistent with previous studies in Ethiopia which indicated that use of routine health information by health workers and health managers is low [28, 30, 31]. Based on systematic review of literature, researchers suggested strategies to improve both data quality and use within routine health information systems in low- and middle-income countries. Among the listed findings, interventions facilitating data availability combined with technology enhancement increased the use of data. Combinations of technology enhancement along with capacity building activities, and data quality assessment and feedback system were recommended for improving data quality [32]. Other studies also showed that routine data quality assessment (RDQA) and intervention based on the identified gaps are good experiences for data quality improvement [33, 34].

The multilevel analysis revealed that there is a significant variation in receiving full immunization service by children background characteristics including religion (lower in Muslims), father’s education (higher in educated) and household wealth status (highest wealth quintile). The lower immunization coverage among Muslims can be linked to lower access to health facilities in emerging regions (pastoral and agro-pastoral areas) in which most residents are Muslims.

Many studies conducted relatively in similar setting are in line with our findings regarding the role of better education and socio-economic status where better parental education and higher wealth has significant contribution for use of immunization service [8, 35]. This is likely due to the fact that both wealthy and educated proportion of the community often have better decision-making capacity in the society and increased health awareness because they have better access to media and other sources of information. Additionally, more wealthy households have better economic ability to pay for services. Even if the service is free for immunization, there are additional costs like transportation especially if the service center is far.

The findings of this study indicated that about 35% of the total variation in children’s full immunization is explained by the difference among health facilities (ICC: 0.35). This implies that working on program management activities including immunization logistics and human resource is very crucial to reduce such differences among health institutions. In line with this finding, the significant role of HIS infrastructure for immunization service uptake is observed. For each additional score of HIS infrastructure availability, the odds of fully immunized children increase by 22% (AOR:1.22; 95% CI: 1.03, 1.44). One of the program inputs for immunization service management is the availability of functional health information system. HIS infrastructure believed to be the limiting factor for the whole health information system of service delivery facilities and program managers to develop automated system. Most facilities have no such functional (automated) systems due to limited HIS infrastructure [16, 36].

### Study limitations

Although, all regions were included, the study findings can be generalizable only to the project sites. The impact of Covid 19 pandemic is not controlled. The study uses three months documentation and report evaluation after COVID pandemic. It may have impact on documentation and reporting. Some of the evidences rely on memory of the respondents which can be affected by recall bias. Finally, in most of composite indices, calculation is done based on series of questions with binary outcomes. These questions have usually provided equal weight which may not be necessarily reflecting the contributions for certain measurements.

## Conclusions

Child immunization coverages were promising in the study areas. However, missing cards & documentation problems affected estimation of valid coverage, especially full immunization. HIS performance is suboptimal. Service user related variables including religion, educational status of father, and wealth index are significantly associated with immunization coverages. HIS infrastructure availability score was significantly associated from HF level variables. Documenting required information and advising mothers to keep immunization cards by health workers, and working to have functional HIS are recommended. Understanding the effect of HIS interventions overtime (trends s of changes) by re-evaluating with similar measurement tools is also recommended.

## Supplementary Information

**Additional file 1**. Operational definitions for selected Health Information System (HIS) variables.

## Data Availability

The datasets used and/or analyzed during the current study are available from the corresponding author but may not be shared until further analysis with the endline data.

## Abbreviations and Acronyms

ANC: Antenatal Care
AOR: Adjusted Odds ratio
BCG: Bacille Calmette-Guérin
CBMP: Capacity Building and Mentorship Program
DPT: Diphtheria Pertussis and Tetanus
EDHS: Ethiopia Demographic and Health Survey
EPHA: Ethiopian Public Health Association
EPI: Expanded Program on Immunization
HepB: Hepatitis B
Hib: Hemophilus influenza type b
HIS: Health Information System
HMIS: Health Management Information System
ICC: Intra-class Correlation Coefficient
IRB: Institutional Review Board
MCV: Measles Containing Vaccine
MOH: Ministry of Health
NCoD: National Classification of Diseases
OPV: Oral Polio Virus
PCV: Pneumococcal Conjugate Vaccine
PRISM: Performance of Routine Information System Management
RHB: Regional Health Bureau
