# Supplementary material for: Contribution of health information system to child immunization services in Ethiopia: baseline study of 33 woredas
Source: BMC Med Inform Decis Mak. 2022 Mar 11;22:64. doi: 10.1186/s12911-022-01796-8 (PMC8913851; doi:10.1186/s12911-022-01796-8)
Supplement: Supplementary file 1 — Additional file 1. Operational definitions for selected Health Information System (HIS) variables. [file 12911_2022_1796_MOESM1_ESM.docx]

**Additional file 1: Operational definitions for selected Health Information System (HIS) variables**

| 1. **HIS resources**   **HIS infrastructure**  This is measured by taking the average score of availability of key HIS infrastructure at facility level. The maximum scores for each facility is 7 (100%) if all key infrastructure exist. The key infrastructures include; a) availability of central MRU, b) availability of shelves, c) availability of Healthnet, d) availability of electricity or generator, e) patient card easily accessible, f) dedicated desk for HMIS, and g) dedicated computer for DHIS2.  **Average eHIS tools availability score**  It was measured by taking the average score of availability of functional eHIS tools including EMR, DHIS2, eCHIS, and HRIS. Each facility will have maximum score of 4 (100%) if all the listed eHIS exist and functional.  **HIS trained workforce**  Percentage of facility staff who have received HIS training (of those who are responsible for performing various HIS tasks). HIS related trainings include: basic HMIS, data quality, data use, data analysis, CHIS, DHIS2, NCOD, integrated (DQ, DU, DHIS2) other related trainings. The staff is considered trained if the person received any one of these trainings over the past one year. This refers to all those staff who are responsible for performing various HIS tasks ranging from data recording to data use.   1. **Data management**   **Average score of data quality control practices**  Measured by taking the average score of health facilities data quality control practice using the following indicators; a) HFs designated person or group in place to review data quality, b) HFs trained all designated staff on HMIS data review and quality control, c) HFs use data quality assessment tools, d) HFs conducted LQAS in the review period, e) HFs maintained records of the LQAs sheets conducted, and f) HFs maintain record of feedbacks given to staff based on data quality assessment. The maximum scores for each facility is 6 (100%) if all indicator present.  **Average score for level of data analysis practices**  It is the average score of health facilities data analysis practice based on the following data analysis indicators; a) aggregated/summary HMIS report - 3 points, b) demographic data on the catchment population of the health facility for calculating coverage -3 points, c) evidence of indicators (e.g., Penta3 coverage) calculated for the health facility catchment within the review three months - 3 points, d) Comparisons between health facility and Woreda/national targets. - 1 point, e) comparisons of data over time (monitoring trends) (e.g., for ANC, Penta3, etc.) - 1 point, f) comparisons of sex-disaggregated data (e.g., for Penta3, HIV testing, provider-initiated counseling and testing [PICT]) -1 point, and g) comparisons of service coverage (e.g. ANC, TT immunization, SBA) - 1 point. The maximum scores for each facility is 13 (100%) if all indicators fulfilled.  **Data visualization practice**  Percentage of facilities that are using raw HMIS data to produce data visuals showing achievements toward targets. Data visuals refers to graphs, tables, maps, etc. showing achievements toward targets (indicators, geographic and/or temporal trends, and situation data).  **Presence of feedback mechanism**  Percentage of facilities confirming receiving feedback on the reported HMIS data from the woreda or higher level.   1. **Data quality**   **Completeness of source document**  Percentage of facilities with completely filled primary source documents, such as registers, patient records, etc. for selected indicators (i.e., source documents contain the data relevant to the selected indicators).  **Timeliness of facility reporting**  Percentage of submitted facility monthly reports (by report type) that are received on time (i.e. by the deadline of reporting).  **Facility reporting accuracy (VF or reporting consistency)**  Percentage of facilities where data recorded in source documents are exactly matching reported data of selected indicator. This is the percentage of facilities that scored verification factor (VF) between 90% - 110% for selected indicators. Agreed tolerance/ acceptability error range by MOH is +/- 10%. I will be over-reporting if < 90% and under-reporting if > 110%.   1. **Use of information**   **Use of routine data for HMIS quality improvement**  It is the average score of health facilities using data to improve HMIS data quality. For each facility, the sum of the scores is calculated based on evidence of Performance monitoring team (PMT) using data to improve HMIS data quality in the following areas (each positive response assigned 1 point); a) discussions on HMIS management, such as data quality, completeness, or timeliness of reporting, b) HMIS related issues identified and prioritized, c) root cause analysis conducted for the prioritized HMIS related issues, d) action plan developed to improving HMIS performance, and e) follow-up actions taken.  **Use of routine data for performance review**  This is the average score of health facilities using data for performance review. For each health facility, the sum of the scores for evidence of using data to review and improve facility performance in the following areas (each positive response assigned 1 point); a) discussed on key performance and quality of care indicators using HMIS data, b) identified and prioritized performance issues/problems, c) Conducted root cause analysis, d) made evidence-based decision, e) developed action plan for improving performance, and f) follow-up actions taken.  **Use of routine data for planning and target setting**  Percentage of health facilities reflect use of HMIS data for planning (annual plan) and target setting.  **Use of routine data to produce analytic report**  Percentage of health facilities with evidence of analytical report production using HMIS data. |
| --- |
